# Prevalence and Predictive Factors of Angle’s Class Malocclusion Asymmetries Without Crossbite in Primary School Children: A Cross-Sectional Study

**DOI:** 10.3390/children12111473

**Published:** 2025-11-01

**Authors:** Marolita Orazi, Maria Grazia Cagetti, Lucia Giannini, Niccolò Cenzato, Cinzia Maria Norma Maspero

**Affiliations:** 1 Department of Biomedical, Surgical and Dental Sciences, University of Milan, 20122 Milan, Italy; 2 Fondazione IRCCS Ca’ Granda Ospedale Maggiore Policlinico, 20122 Milan, Italy

**Keywords:** malocclusion, Angle’s dental class, occlusal asymmetry, pediatric orthodontics, oral breathing, craniofacial development, etiology, predictive factors

## Abstract

Background: Angle’s dental class asymmetries not associated with crossbite are malocclusions that are often underestimated in pediatric patients. However, they may be associated with alterations in the development of the stomatognathic system. Objective: The objective of this study was to evaluate the prevalence of Angle’s class asymmetries without crossbite in primary-school-aged children and to investigate possible associations with perinatal, clinical, and functional variables. Materials and Methods: This cross-sectional observational study analyzed a sample of 391 children aged 6 to 11 years, attending a primary school in the metropolitan area of Milan, Italy. Data were systematically collected through both clinical examination and patient history, with the aim of identifying significant correlations with the occurrence of dental asymmetries in the absence of crossbite. Results. The results revealed a higher prevalence of occlusal asymmetries associated with factors such as oral breathing, low tongue posture, type of delivery, formula feeding, and systemic diseases during the first three years of life. Advanced carious lesions and inclination of the occlusal plane were significantly associated with asymmetry. Conclusions: The study highlights the importance of early diagnosis and a multidisciplinary approach to prevent malocclusions and complex craniofacial dysfunctions later in life.

## 1. Introduction

Malocclusions represent morpho-functional alterations of the stomatognathic system which, if not diagnosed and promptly treated during developmental age, may be associated with impaired craniofacial growth and less harmonious skeletal development [1,2].

Asymmetries in Angle’s classification without crossbite are often overlooked in children. Yet they may negatively affect craniofacial growth, with long-term consequences for facial aesthetics and masticatory function [3].

The concept of dental occlusion was first introduced by Edward H. Angle in 1890, who classified inter-arch relationships into three occlusal classes based on the anteroposterior position of the permanent first molars. He identified Class I as normal (neutro-occlusion), distinguishing it from Class II (distal positioning of the lower molars) and Class III (mesial positioning of the lower molars).

Asymmetries in Angle’s classification refer to all malocclusions in which there is a discrepancy in molar occlusion between the right and left sides.

Numerous studies have suggested that the interplay between genetic and epigenetic environmental factors plays a significant role in the etiology of craniofacial asymmetries [4,5,6].

Moss, in his Functional Matrix Theory, emphasized the role of functions such as mastication, breathing, and posture in modulating bone growth [5]. Similarly, P. Planas underscored the importance of alternate bilateral mastication as a key stimulus for the symmetric development of the maxilla and mandible.

Recent studies, including those by M.J. Deshayes, have further reinforced the concept that craniofacial biodynamics, driven by the synchronized flexion–extension of the occipital and sphenoid bones, is essential for maintaining morphological and functional balance of the face [7].

Dysfunctions such as oral breathing, low tongue posture, and artificial feeding have been identified as potential predisposing factors for asymmetric development of the dental arches [8,9,10].

However, scientific literature has paid limited attention to dental asymmetries in the absence of crossbite [9,10]. These conditions may in fact represent early signs of potential asymmetric growth, with possible long-term associations involving the occlusal plane, the temporomandibular joint (TMJ), and body posture.

The aim of the present study was to analyze the prevalence of Angle’s dental class asymmetries not associated with crossbite in school-aged children and to investigate possible correlations with perinatal and early childhood anamnesis (first three years of life), clinical findings, and functional variables. The overarching goal is to raise clinical awareness of these conditions and to support the early identification of occlusal imbalances during developmental age.

## 2. Materials and Methods

### 2.1. Study Design and Ethical Considerations

This was a descriptive cross-sectional observational study conducted at a primary school located in the metropolitan area of Milan, Italy. The design was chosen to estimate the prevalence of Angle’s class asymmetries without crossbite and to investigate their associations with perinatal, clinical, and functional factors. The study adhered to the principles outlined in the Declaration of Helsinki and received approval from the Ethics Committee (Institutional Review Board) of the Fondazione IRCCS Ca’ Granda, Ospedale Maggiore Policlinico (Protocol No. 421, approved on 9 March 2024). The study was designed and reported in accordance with the STROBE (Strengthening the Reporting of Observational Studies in Epidemiology) guidelines for cross-sectional studies.

### 2.2. Study Size

The sample size was determined by the available number of children in the school (grades 1–5). Simple random sampling was used to yield the intended sample size of 391 children. No specific sample size calculation was performed a priori; nevertheless, with this sample, it was possible to estimate a prevalence of asymmetry of approximately 30% with a precision of ±5 at the 95% confidence level.

### 2.3. Sample Selection

The sampling frame was all grade 1–5 children of the study primary school (6/11 years old). The school administration provided a list of eligible students, which was de-identified.

We constructed a computerized simple random ordering (without replacement) of the roster and then invited families in the order drawn randomly until reaching the targeted sample size, given receipt of written parental consent and, on the examination day, child assent.

No other specific inclusion or exclusion criteria were used other than consent/assent and presence on the day offered. A total of 391 children (192 males and 199 females) aged between 6 and 11 years were randomly selected.

### 2.4. Data Collection Protocol

Data collection was structured into three main phases:

1. Medical and dental history

A standardized medical history questionnaire was completed by the parents, including items related to:

I. Type of delivery (eutocic or dystocic)
II. Type of feeding (breastfeeding or formula feeding)
III. Presence of systemic diseases during the first three years of life
IV. Oral hygiene habits at home

Birth complications were defined as dystocic delivery, caesarean section, forceps or vacuum-assisted delivery or perinatal respiratory distress requiring medical support.

2. Preliminary Interviews

Each child participated in two short interviews. The first focused on dietary habits, while the second explored past or ongoing orthodontic treatments.

We interviewed children in order to get some background information about their dietary habits and whether they had ever been treated for orthodontic problems.

These data were gathered in a simple format to supplement with additional information from the questionnaire for parents. We know that this method involves the handicap of dependency on parental reports for information about dietary habits.

3. Intraoral Examination and Orthodontic Evaluation

Two separate intraoral examinations were performed by trained dental professionals. The following clinical parameters were recorded:

- Intraoral Clinical Assessment:
  ▪ Dental formula;
  ▪ Presence of enamel defects (DDE), based on the DDE Index;
  ▪ Presence and severity of carious lesions, based on the ICDAS Index;
  ▪ Plaque Index (Silness and Löe).
- Orthodontic Assessment:
  ▪ Type of dentition (primary, mixed, or permanent);
  ▪ Presence of crossbite;
  ▪ Presence of asymmetry in Angle’s dental classification;
  ▪ Coincidence of midlines (dental and labial frenula);
  ▪ Inclination of the occlusal plane;
  ▪ Functional assessment, including evaluation of oral breathing, tongue posture, and lip competence.

### 2.5. Diagnostic Tools and Criteria

- Molar and canine relationships were assessed using Angle’s classification in intercuspal position with the child seated upright and teeth in maximum intercuspation.
- Dental class asymmetries were defined as clinically detectable differences in molar or canine relationships between the right and left sides.
- ICDAS criteria were used to classify the severity of cavity lesions by two calibrated examiners.
- Occlusal plane inclination was assessed via direct clinical observation.
- Oral breathing was diagnosed using the Gudin Nasal Reflex Test and 1 min rest observation (classified as present only if both were positive).
- Low tongue posture was confirmed through direct observation (evaluated at rest and during swallowing; classified as present if the tongue did not contact the incisive papilla at rest and/or showed anterior position during swallow).

### 2.6. Examiner Training and Reliability

The dental examinations were conducted by two trained and calibrated dental examiners, using a standardized calibration process. Agreement was calculated in terms of Cohen’s κ and percent agreement, with 95% confidence intervals. Differences in the primary dataset were resolved by consensus discussion.

### 2.7. Bias Selection

Selection bias may have occurred due to voluntary parental consent and the requirement for attendance on the day of examination. This potential limitation has been acknowledged in the Discussion.

### 2.8. Handling of Missing Data

Missing data were minimal (<5% for all variables). Cases with missing information for specific variables were excluded only from the relevant analyses, and the number of observations used for each test is reported in the corresponding tables.

### 2.9. Statistical Analysis

All data were entered into a single anonymized Excel spreadsheet; each participant was assigned a unique identification code to ensure confidentiality.

Descriptive statistics included means and standard deviations for continuous variables and frequencies with 95% confidence intervals for categorical variables. Associations between dental asymmetry and explanatory variables were evaluated through a bivariate analysis using Chi-square tests or non-parametric tests, as appropriate. All analyses were two-tailed with a significance threshold of 0.05.

These analyses were performed to explore associations between Angle’s dental class asymmetries (in the absence of crossbite) and the following variables:

- Perinatal factors (e.g., delivery complications, feeding method)
- Breathing conditions during the first three years of life
- Early loss or presence of advanced carious lesions in the second primary molars
- Dysfunctional oral habits such as oral breathing, low tongue posture, and lip incompetence

We report all prevalence estimates with 95% confidence intervals (Wilson method).

## 3. Results

### 3.1. Sample Characteristics

The study sample consisted of 391 children (192 males and 199 females) aged between 6 and 11 years (mean age: 7.9 ± 1.3 years). The distribution by age and sex is shown in Table 1.

### 3.2. Height and Weight

The mean height was 129.8 cm (SD: 12.3), and the mean weight was 28.9 kg (SD: 7.2 kg). No statistically significant differences were found between males and females (*p* > 0.05 for all variables).

### 3.3. Type of Dentition

Most children (96.6%) presented with mixed dentition; 1.5% had primary dentition, and 1.7% had permanent dentition.

### 3.4. Angle’s Class Asymmetries and Crossbite

**Figure 1:**
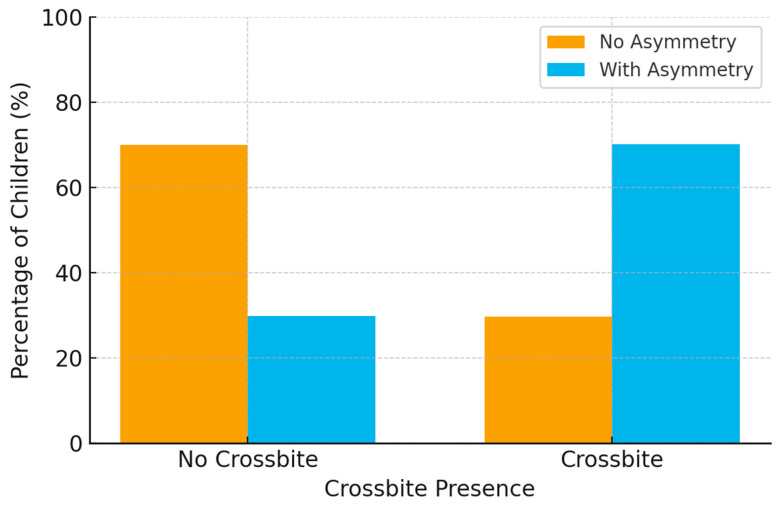
Prevalence of Angle’s Class Asymmetry in Children With and Without Crossbite.

Among the children, 9.4% had a complete crossbite. Of these, 70.2% exhibited Angle’s class asymmetry. Among those without crossbite (90.5% of the sample), 29.9% presented asymmetry. (Table 2 and Figure 1).

### 3.5. Associations with Anamnestic and Functional Variables in Children Without Crossbite

Significant associations were found between dental asymmetries and several perinatal and functional variables (Table 3):

- Dystocic delivery: 37% among asymmetric children (*p* < 0.05)
- Formula feeding: 54% among asymmetric children (*p* < 0.05)
- Systemic diseases in the first 3 years: 22% of asymmetric cases (*p* < 0.01)

The most frequent birth complications were caesarean section and assisted vaginal deliveries, while a smaller part of children had experienced neonatal respiratory distress.

#### Associated Occlusal Parameters

Angle’s class asymmetries were also found to be significantly associated with specific dento-occlusal factors in children without crossbite.

An inclination of the upper occlusal plane was observed in 38.6% of asymmetric subjects, compared to 19.3% of symmetric individuals (*p* < 0.001) (Table 4) Among children with an inclined plane, 68.2% showed non-coincidence of the dental midlines, and 70.1% showed non-coincidence of the labial frena (*p* < 0.001 for both) (Table 5).

Early loss of primary canines (teeth 53, 63, 73, 83) was observed in 28.7% to 29.1% of subjects with asymmetry (*p* < 0.01) (Table 6).

24.0% of the sample experienced early loss of at least one second primary molar, with the highest frequency involving teeth 75 (2.8%) and 85 (3.1%). Although not statistically significant, early loss of tooth 75 (*p* = 0.101) and tooth 85 (*p* = 0.153) suggests a potential association with dental asymmetry (Table 7).

Advanced carious lesions in second primary molars (ICDAS 6) were found in 29.9% of children with asymmetry, with statistically significant associations for teeth 65 and 85 (*p* < 0.029) (Table 8).

ICDAS 6 lesions were more frequently found in children with an inclined occlusal plane (54.0–58.3%) than in those with a neutral plane. The *p*-values for teeth 55 and 65 (*p* = 0.104, *p* = 0.153) suggest that advanced caries in upper second primary molars may contribute to occlusal plane inclination (Table 9).

Functional analysis showed a higher prevalence of Angle’s class asymmetry in children with oral breathing (32.4%), low tongue posture (32.3%), and lip incompetence (31.4%). In particular, low tongue posture showed a trend toward statistical significance (*p* = 0.056) (Table 10).

### 3.6. Orthodontic Treatments

A total of 88.4% of children with dental asymmetry (89 out of 106) had never undergone orthodontic treatment. Moreover, 42.8% of those currently wearing an appliance (9 out of 21) did not present any asymmetry (Table 11).

## 4. Discussion

Although the findings do not fully cover all aspects of the initial objective, they provide supportive evidence for the hypothesis that Angle’s dental class asymmetries without crossbite represent a clinically significant form of malocclusion. These asymmetries are frequently linked to early systemic conditions, dysfunctional habits, and improper feeding or oral hygiene practices. With a prevalence of 27% in the study sample, such asymmetries are not rare at developmental age, and the fact that they often go undiagnosed underlines the need for greater clinical and preventive attention. Comparable studies are scarce, as most epidemiological reports focus on crossbite or sagittal discrepancies rather than on Angle’s class asymmetries without crossbite. Nevertheless, existing data suggest variable prevalence across populations. For instance, Grippaudo et al. reported asymmetry in approximately 20% of Italian schoolchildren, whereas Srivastava et al. noted a prevalence ranging from 15% to 25% depending on diagnostic criteria [11,12].

Lombardo et al., in a 2020 systematic review of global malocclusion prevalence, concluded that occlusal asymmetries are underreported but may represent a substantial proportion of non-crossbite malocclusions with a prevalence ranging from 15% to 30% depending on diagnostic criteria. These findings suggest that the 27% prevalence observed in our sample is within the upper range of values described in other populations [9].

A trend toward an association between oral breathing and Angle’s class asymmetry was observed, although it did not reach statistical significance in our sample (*p* = 0.274), whereas previous studies have reported significant associations [11,12,13,14]. Oral breathing has been reported to be associated with altered tongue posture and orofacial muscle tonicity, which in turn may contribute to imbalances in dental arch development [9].

Low tongue posture, observed in 32.3% of asymmetric subjects, also emerged as a relevant etiological factor. As described by Planas and subsequent authors, resting tongue position influences the remodeling of maxillary and mandibular structures and may contribute to the development of asymmetries, particularly when associated with unilateral mastication [6,8].

The link between formula feeding and occlusal alterations is supported by numerous studies attributing a protective role to breastfeeding in the physiological and symmetrical development of the stomatognathic system [15].

The correlation between Angle’s class asymmetry and the inclination of the upper occlusal plane was highly significant. As Deshayes proposed in his theory of craniofacial dynamics, an inclined occlusal plane may be associated with alterations in the cranial base flexion–extension mechanism [7]. A non-symmetric occlusal plane may reflect or even contribute to asymmetric growth of the craniofacial structures and to mandibular functional imbalances.

The early loss of second primary molars did not show a statistically significant association with Angle’s class asymmetry in our sample, but a potential trend was observed, suggesting that this factor may warrant further investigation in larger populations. The premature loss of deciduous elements (along with inadequate functional stimulation) has been associated with disturbances in eruption guidance and occlusal balance [13].

Data analysis also revealed that 88.4% of children with dental asymmetries (89 out of 106) had never received orthodontic treatment, despite a clear clinical indication. Among those currently undergoing treatment, 57.1% (12 out of 21) still showed asymmetries, possibly due to delayed intervention, ineffective appliance design, or unfavorable biological response. The delayed recognition of these malocclusions may result from underestimation by both parents and clinicians, as well as a lack of awareness regarding the need for early treatment. Additionally, socioeconomic barriers may limit access to appropriate orthodontic care [16,17,18,19,20,21].

A multidisciplinary approach may be beneficial, involving the dentist, orthodontist, speech therapist, pediatrician, and, when needed, a physiotherapist. Early diagnosis and timely correction of dysfunctions represent the most effective strategy for preventing more complex skeletal malocclusions in adulthood [22,23,24].

A large proportion of children with dental asymmetries had not received any orthodontic intervention, despite the clinical significance. These results confirm the high prevalence of malocclusion in the pediatric population and underscore the importance of early and multidisciplinary intervention to prevent long-term aesthetic and functional consequences.

Timely diagnosis, caries prevention, occlusal balance control, and monitoring of neuromuscular function are therefore key strategies. In this context, the pediatric dentist plays a central role in intercepting these anomalies and guiding the child toward a proper therapeutic path [25].

Although our results are in agreement with findings of the earlier studies, alternative interpretations need to be evaluated: the reported associations with oral breathing or early caries may may indicate sharedunderlying susceptibilities rather than direct causal mechanisms. The observed results showed that some researchers obtained low or no correlation between feeding methods, functional habits, and occlusal asymmetries, which suggests that studies on this topic are diverse. Our findings should be read as exploratory and hypothesis-generating until replicated in longitudinal cohorts.

### 4.1. Limitations and Potential Bias

This study has some limitations. The cross-sectional nature of the design is appropriate for estimating prevalence, but it is not helpful in establishing temporal relationships or permitting causal inference. Findings should therefore be interpreted as associations only, and longitudinal studies would be needed in order to verify whether observed associations are causal ones for the development of Angle’s class asymmetries without crossbite.

Another limitation is about the sample selection: despite the use of a simple random sample, participation was voluntary and based on parental consent and attendance on examination day was mandatory. Consequently, self-selection and non-response could have led to selection bias. Multiple bivariate analyses were performed, which may increase the risk of type I error; no formal correction for multiple comparisons was applied, and this should be considered when interpreting the results.

Socioeconomic status, dietary habits, and access to dental care were not assessed, so residual confounding by these factors cannot be excluded.

A further limitation is that orthodontic treatment history were reported directly by the children, which may be less reliable than parental reporting. In addition, although ICDAS II is the updated version, we used the original ICDAS criteria, as examiners had previously been calibrated on this system.

Regarding bias, information bias was minimized by examiner calibration, but some misclassification cannot be excluded. Furthermore, residual confounding from unmeasured variables is possible.

### 4.2. Generalizability

Because the sample was drawn from a single primary school in the Milan metropolitan area, caution is warranted when generalizing these results to other populations. Nevertheless, the random sampling procedure and the relatively large sample size give strength to the internal validity of the findings. These findings may provide useful insights for the interpretation of prevalence estimates and for understanding the magnitude of the associations observed.

## 5. Conclusions

In this cross-sectional study, Angle’s class asymmetries without crossbite were common in school-aged children. Statistically significant associations were observed with occlusal plane inclination and advanced carious lesions in specific teeth. Other factors, such as oral breathing, low tongue posture, early tooth loss, and perinatal conditions, showed only non-significant trends and should be considered exploratory. These findings suggest a complex interaction between skeletal, occlusal, and neuromuscular factors in the etiology of malocclusion and underline the importance of an early diagnosis, while further longitudinal studies are needed to confirm causality.

## Figures and Tables

**Table 1:** Mean values of age, weight, and height by sex.

Variable	Mean (F)	SD (F)	Mean (M)	SD (M)	*p*-Value
Age	7.9	1.3	7.8	1.3	>0.05
Weight	28.6 kg	7.0 kg	29.3 kg	7.4 kg	>0.05
Height	129.8 cm	12.7 cm	129.7 cm	12.0 cm	>0.05

**Table 2:** Presence of Angle’s class asymmetry in children with and without crossbite.

Crossbite	No Asymmetry (*n*)	With Asymmetry (*n*)	Total (*n*)	No Asymmetry (%)	With Asymmetry (%)	% of Sample
No	248	106	354	70.0%	29.9%	90.5%
Yes	11	26	37	29.7%	70.2%	9.4%
Total	259	132	391	66.2%	33.7%	100%

**Table 3:** Association between birth complications, breastfeeding, respiratory diseases and Angle’s class asymmetry in children without crossbite.

Birth Complications	No Asymmetry (*n*)	With Asymmetry (*n*)	Total (*n*)	No Asymmetry (%)	With Asymmetry (%)	*p*-Value
No	159	58	217	73.2%	26.7%	0.466
Yes	89	48	137	64.9%	35.0%	0.326
Total	248	106	354	70.0%	29.9%	0.096
Breastfeeding	No Asymmetry (*n*)	With Asymmetry (*n*)	Total (*n*)	No Asymmetry (%)	With Asymmetry (%)	*p*-value
No	54	27	81	66.6%	33.3%	
Yes	192	79	271	70.8%	29.1%	
Total	246	106	352	69.8%	30.1%	0.472
Respiratory Diseases	No Asymmetry (*n*)	With Asymmetry (*n*)	Total (*n*)	No Asymmetry (%)	With Asymmetry (%)	*p*-value
No	208	80	288	72.2%	27.7%	0.063
Yes	40	26	66	60.6%	39.3%	0.063
Total	248	106	354	70.0%	29.9%	0.063

**Table 4:** Association between occlusal plane inclination and Angle’s class asymmetry in children without crossbite.

Occlusal Plane	No Asymmetry (*n*)	With Asymmetry (*n*)	Total (*n*)	No Asymmetry (%)	With Asymmetry (%)	*p*-Value
Neutral	129	31	160	80.6%	19.3%	<0.001
Inclined Right	53	30	83	63.8%	36.1%	<0.001
Inclined Left	66	45	111	59.4%	40.5%	<0.001
All Inclined	119	75	194	61.3%	38.6%	<0.001

**Table 5:** Midline coincidence, labial frenum coincidence and occlusal plane inclination in children without crossbite.

Midline Coincidence	Neutral Plane (*n*)	Neutral (%)	Inclined Plane (*n*)	Inclined (%)	Total	*p*-Value
No	40	31.7%	86	68.2%	126	<0.001
Yes	208	91.2%	20	8.7%	228	<0.001
Total	248	70.0%	106	29.9%	354	
Frenum Coincidence	Neutral Plane (*n*)	Neutral (%)	Inclined Plane (*n*)	Inclined (%)	Total	*p*-value
No	34	29.8%	80	70.1%	114	<0.001
Yes	214	89.1%	26	10.8%	240	<0.001
Total	248	70.0%	106	29.9%	354	

**Table 6:** Association between early loss of primary canines and Angle’s class asymmetry in children without crossbite.

Tooth	No Asymmetry (*n*)	With Asymmetry (*n*)	Total (*n*)	No Asymmetry (%)	With Asymmetry (%)	*p*-Value
53	170	69	239	71.1%	28.8%	0.357
63	168	69	237	70.8%	29.1%	0.822
73	162	66	228	71.0%	28.9%	0.717
83	164	66	230	71.3%	28.7%	0.451

**Table 7:** Early loss of second primary molars and association with Angle’s class asymmetry in children without crossbite.

Tooth	No Asymmetry (*n*)	No (%)	With Asymmetry (*n*)	With (%)	Total (*n*)	Total Sample Loss (%)	*p*-Value
55	237	70.7%	98	29.2%	335	2.2%	0.234
65	236	70.2%	100	29.7%	336	1.6%	0.747
75	236	71.0%	96	28.9%	332	2.8%	0.101
85	233	71.0%	95	28.9%	328	3.1%	0.153

**Table 8:** Association between ICDAS 6 lesions in second primary molars and Angle’s class asymmetry.

ICDAS 6	No Asymmetry (*n*)	No (%)	With Asymmetry (*n*)	With (%)	Total (*n*)	*p*-Value
55	241	70.0%	103	29.9%	344	0.997
65	243	71.0%	99	28.9%	342	0.029
75	240	70.1%	102	29.8%	342	0.794
85	243	71.0%	99	28.9%	342	0.029

**Table 9:** ICDAS 6 lesions and occlusal plane inclination in children without crossbite.

ICDAS 6 Index	Neutral Plane (*n*)	Inclined Plane (*n*)	Total (*n*)	Neutral (%)	Inclined (%)	*p*-Value
55	158	186	344	45.9%	54.0%	0.104
65	157	185	342	45.9%	54.0%	0.153
75	156	186	342	45.6%	54.3%	0.401
85	155	187	342	45.3%	54.6%	0.803

**Table 10:** Functional findings and association with Angle’s class asymmetry in children without crossbite.

Functional Exam	No Asymmetry (*n*)	With Asymmetry (*n*)	Total (*n*)	No Asymmetry (%)	With Asymmetry (%)	*p*-Value
Oral Breathing—Yes	127	61	188	67.5%	32.4%	0.274
Tongue Posture—No	60	16	76	78.9%	21.0%	0.056
Tongue Posture—Yes	188	90	278	67.6%	32.3%	0.056
Lip Competence—No	39	10	49	79.5%	20.4%	0.116
Lip Competence—Yes	209	96	305	68.5%	31.4%	0.116

**Table 11:** Previous orthodontic treatment, current orthodontic treatment and presence of Angle’s class asymmetry.

Treatment History	No Asymmetry (*n*)	No Asymmetry (%)	With Asymmetry (*n*)	With Asymmetry (%)	Total (*n*)	Total (%)	*p*-Value
No	224	71.5%	89	28.4%	313	88.4%	0.087
Yes	24	58.5%	17	41.4%	41	11.5%	
Currently Wearing Appliance	No Asymmetry (*n*)	No Asymmetry (%)	With Asymmetry (*n*)	With Asymmetry (%)	Total (*n*)	Total (%)	*p*-value
No	236	70.8%	97	29.1%	333	94.0%	0.183
Yes	12	57.1%	9	42.8%	21	5.9%	

Please note that each *p*-value corresponds to an independent chi-square test performed for the specific variable.

## Data Availability

The datasets generated and analyzed during the current study are not publicly available due to privacy restrictions involving minors but are available from the corresponding author in anonymized form upon reasonable request and following the signature of a data-sharing agreement in compliance with institutional and ethical guidelines. The English-translated version of the medical questionnaire used in this study is available from the authors upon reasonable request.

## Abbreviations

DDE	Developmental Defects of Enamel
ICDAS	International Caries Detection and Assessment System
RR	Risk Ratio
CI	Confidence Interval
κ	Cohen’s kappa
*p*	*p*-value (unadjusted)
